# Effects of Agro-Industrial Byproduct-Based Diets on the Growth Performance, Digestibility, Nutritional and Microbiota Composition of Mealworm (*Tenebrio molitor* L.)

**DOI:** 10.3390/insects13040323

**Published:** 2022-03-25

**Authors:** Ana Montalbán, Cristian Jesús Sánchez, Fuensanta Hernández, Achille Schiavone, Josefa Madrid, Silvia Martínez-Miró

**Affiliations:** 1 Department of Animal Production, Faculty of Veterinary Science, Regional Campus of International Excellence “Mare Nostrum”, University of Murcia, Espinardo, 30100 Murcia, Spain; ana.montalban1@um.es (A.M.); nutri@um.es (F.H.); alimen@um.es (J.M.); silviamm@um.es (S.M.-M.); 2 Department of Veterinary Sciences, University of Turin, 10095 Grugliasco, Italy; achille.schiavone@unito.it

**Keywords:** insect larvae, agro-industrial waste, feed formulation, feed conversion ratio, gut microbiota

## Abstract

**Simple Summary:**

The mealworm (*Tenebrio molitor*) is among the species considered to have the greatest potential to become a valid source of protein for consumption. In this study, mealworm larvae were reared on diets formulated with agro-industrial byproducts to evaluate the impact on their growth performance, digestibility, and nutritional composition as well as on the modulation of their gut microbiota profile. Depending on the diet composition, improvements were observed in the conversion rate (for diets with a smaller variety of byproducts and a higher starch content) or in the growth (for diets with a greater variety of byproducts and a higher protein content) of larvae. The diets also had repercussions for the gut microbiota profile and composition of the larvae. These results improve knowledge of the effects of byproducts used in mealworm larval rearing, which could contribute to their potential use in industrial-scale mealworm production.

**Abstract:**

The aim of this study was to evaluate the effects of agro-industrial byproduct-based diets on the productive parameters, digestibility, insect composition, and gut microbiota of mealworm (*Tenebrio molitor*) larvae. Three formulations corresponding to three different levels of starch and protein were tested: one formulation based on bread remains and brewer’s yeast, representing a diet of high starch (61.1%) and low crude protein (18.5%) (HS-LP); and two formulations in which an additional four byproducts (courgette (*Cucurbita pepo*) remains, tigernut (*Cyperus scelentus*) pulp, brewer’s spent grains, and rice straw) were incorporated in different proportions, consistent with a diet of both moderate starch (29.8%) and crude protein (21.0%) (MS-MP); and another corresponding to a diet of low starch (20.0%) and high crude protein (26.3%) (LS-HP). A total of 1920 young larvae (average weight = 0.65 mg per larva) were used in this study. The larvae were randomly distributed into 16 replicates per treatment (boxes of 22.5 cm × 14.0 cm × 4.75 cm). Ten replicates for the growth performance–digestibility trial and six replicates for the complementary trial to determine uric acid levels in the frass were assigned per treatment. For growth performance, the diets were administered ad libitum during the experiment. The average number of days for the larvae to start pupating was lower in those reared on the HS-LP and LS-HP diets (88.90 and 91.00 days, respectively) than those on the MS-MP diet (120.09 days) (*p* < 0.001). The final individual weight was higher (*p* < 0.001) in larvae of the LS-HP group (168.69 mg) compared to those of the other groups (100.29 and 112.99 mg for HS-LP and MS-MP, respectively). However, the feed conversion ratio was better (*p* < 0.001) in the HS-LP group with the lowest value (1.39 g/g), with dry matter digestibility being the highest for the same diet (70.38%) (*p* < 0.001). Mealworms reared on LS-HP and MS-MP diets had a higher crude protein content than those reared on the HS-LP diet (*p* = 0.039). The most abundant phyla in the gut microbiota of larvae were Tenericutes, Proteobacteria, and Firmicutes, with their abundance depending on the rearing substrate. The representation of Tenericutes phylum was higher (*p* < 0.05) in the mealworms reared on MS-MP and HS-LP diets, whereas Proteobacteria and Cyanobacteria were higher in abundance (*p* < 0.001) in the insects reared on LS-HP. In conclusion, the larval growth, digestibility, insect composition, and gut microbiota of *Tenebrio molitor* were found to depend on the composition of the administered diet, and the results suggest great potential for the use of agro-industrial byproducts in their rearing and production.

## 1. Introduction

Insects have been identified as a potential solution in the search for alternative protein sources for animal nutrition [1,2], being considered a new and sustainable dietary protein for consumption by both humans and farm animals [3]. The attraction of using insects in livestock feeds is based on their low-impact production, which involves lower energy costs, less land area utilization, a lower environmental footprint, and lower related CO_2_ emissions compared with traditional protein sources [4]. Additionally, the farming of insects could potentially have a positive environmental impact due to their consumption of agricultural wastes and byproducts [3]. However, the use of insects in feeds is relatively new in Europe, and a significant gap exists in the production chain between the demand for insects and the current capacity of various actors to supply them. Feed processors would like to use insects as ingredients, but only if continuous quantities of a predefined quality of insects can be supplied; however, insect farms cannot currently produce the required volumes at a standard quality. As a result, the cost of insect protein is not yet competitive in relation to traditional protein sources such as soybean or fish meal [2]; therefore, efforts are needed to ensure insect feeding practices become more efficient and less expensive. In addition, the European Commission recently published Regulation (EU) 2021/1372 [5], which amended the EU’s feed ban regulation to authorize the use of processed animal proteins derived from insects in poultry and pig feed, with the hope of helping create a more sustainable feed chain.

A variety of insect species are currently being proposed for feeds; among them, the larvae of yellow mealworm (*Tenebrio molitor* (TM)) and black soldier fly (*Hermetia illucens*) have the highest potential for large-scale production in the EU [2]. The nutritional properties of TM larvae are also similar to those of soybean and fish meal, and they can be farmed on a variety of substrates and under a wide range of temperatures, which makes them especially suitable in the climate change era [2]. Methods for TM mass-rearing in captivity vary, but most of use wheat bran or wheat flour and brewer’s yeast to create an optimal diet [6]. However, these substrates can be used directly as feed ingredients in farm animal diets; thus, other alternative feeding sources should be found.

Most recently, efforts have been made regarding the valorization of agricultural wastes (e.g., maize stover, carrot, orange, and cabbage side-stems) and byproducts of the agro-industry (beer brewing, bread/cookie baking, potato processing, and bioethanol production) by utilizing them as substrates for rearing TM larvae [6,7,8]. Most of this kind of waste is high in fiber content and therefore largely indigestible by non-herbivorous farm animals but suitable for raising insects. There has been much speculation about TM owing to its ability to adapt to different substrates [1] as a result of its capability to break down cellulose [9], which is a common component in potential sustainable rearing substrates obtained from agricultural waste. However, little is known about this insect species’ specific metabolic response to low-quality feedstocks, although there is growing evidence that its gut microbiota plays an important role in adaption with regard to aspects of digestion, nutrition, defense, reproduction, and metabolism [10,11]. Consequently, the role of the insect gut microbiota in the digestive process needs to be tested. In particular, the ability of the insect microbiota to adapt to different agricultural waste and byproduct mixtures and its impact on larval growth performance and nutritional properties needs to be elucidated. Thus, within the scope of applied research, the aim of this study was to test diets based on byproducts with different levels of starch and protein and evaluate their effects on the performance, digestibility, and gut microbiota of *Tenebrio molitor* larvae.

## 2. Materials and Methods

The study was performed at the Department of Animal Production, University of Murcia (Murcia, Spain). 

### 2.1. Diet Preparation

The diets were prepared using agro-industrial byproducts with different chemical compositions (Table 1): courgette (*Cucurbita pepo*) byproduct, consisting of tails and discards resulting from industrial preparation; tigernut (*Cyperus scelentus*) pulp, resulting from grinding, soaking, and pressing for the artisanal production of tigernut milk; brewer’s spent grains, consisting of starch, germ, and hull residues of barley grain; brewer’s yeast; bread remains; and rice straw. Three formulations corresponding to three diets characterized by different levels of starch and protein were tested: one formulation was based on bread remains and brewer’s yeast, corresponding to a diet of high starch (61.1%) and low crude protein (18.5%) (HS-LP), following a similar experimental design to that of van Broekhoven et al. [7] and Stull et al. [6]; and two formulations in which all byproducts were used but incorporating them in different proportions, obtaining a diet of both moderate starch (29.8%) and crude protein (21.0%) (MS-MP); and another corresponding to a diet of low starch (20.0%) and high crude protein (26.3%) (LS-HP) (Table 2). The MS-MP and LS-HP diets had a higher fiber and ether extract content compared to HS-LP. For the preparation of the diets, the ingredients were dried at 60 °C to a constant weight and ground until particles were around 2 mm in diameter, then dosed and mixed in different proportions for each diet using a mixer and pelleted into pellets of 5 mm in diameter.

### 2.2. Insect Rearing

A total of 1920 young larvae of mealworms (*Tenebrio molitor* L. 1785; Coleoptera: Tenebrionidae) provided by the Department of Biology, University of Murcia (Murcia, Spain), were used. The larvae were randomly distributed across 16 boxes for treatments. The boxes for each treatment were randomly assigned: 10 replicates were used for the performance–digestibility trial, and 6 replicates for the uric acid trial (complementary test). The larvae were separated from the breeding substrate (vegetal substrate) 2 days after hatching. For each replicate, a total of 40 freshly hatched larvae were transferred to a plastic box (22.5 cm × 14.0 cm × 4.75 cm) using a plastic water dispenser to provide humidity. The environmental temperature was maintained at 25 ± 3 °C with 50–60% relative humidity.

For the performance–digestibility trial, the feed was administered ad libitum and supplemented weekly if needed. The amount of added feed was weighed each time. For each replicate, the mealworm harvest was set when the first pupa appeared [12,13]. After harvesting, the larvae were separated from the remaining substrate (refusal feed and frass), weighed, and killed by freezing at −20 °C. Then, the larvae were pooled for 2 replicates in order to obtain 5 samples per treatment for analysis. The refused substrate (refusal feed and frass) was also collected, weighed, and frozen at −20 °C for further analysis. Mortality was recorded daily. 

A uric acid complementary assay was conducted to estimate the intake and diet digestibility [7]. This method allows determining the concentration of uric acid in the excreta depending on the type of diet consumed in such a way that uric acid can be used as an indicator to estimate the amount of frass and the refusal feed in the remaining substrate in the performance–digestibility trial, which also allows the intake and digestibility to be estimated. 

For the uric acid trial, a restricted quantity of feed (5 g) was offered in 6 replicates for each type of diet in order to ensure that the larvae would consume the total amount offered. To check that the diet was entirely consumed, the larvae were weighed every 15 days. When the weight increase stopped, it was considered that the feed was consumed, and the substrate was composed of pure frass. The frass was stored at −20 °C for further uric acid determination. 

### 2.3. Chemical Composition

#### 2.3.1. Proximate Composition 

Dry matter (DM) in byproducts, feeds, refusal substrates, and excreta were determined by drying at 60 °C for 48 h. The DM content in the mealworm larvae was determined by lyophilization.

The byproduct, feed, and larva samples were ground in a laboratory mill to pass through a 1 mm sieve (RETSCH ZM 200 Ultra Centrifugal Mill; RETSCH, Hann, Germany). These samples were analyzed using the procedures of the Association of Official Analytical Chemists (AOAC) [14] for crude protein (CP) (method 2001.11) and ether extract (EE) (method 920.39). For ash determination, samples were ashed at 550 °C for 4 h in a muffle furnace. In the byproduct and feed samples, the starch content was measured polarimetrically using the official Spanish analytical method [15]. The procedures described by Van Soest et al. [16] were used to determine the neutral detergent fiber (NDF) and acid detergent fiber (ADF), with analysis of acid detergent lignin (ADL) through the solubilization of cellulose with 72% H_2_SO_4_.

#### 2.3.2. Uric Acid

For uric acid determination, the method reported by Marquardt [17] based on UV–Vis spectrophotometry (UNI-CAN UV–Vis Spectrometry, Helios Gamma, Loughborough, UK) was followed. In brief, 50 mg of either rejected substrate or pure frass was extracted in 100 mL of glycine buffer solution at pH 9.3 and incubated for 1 h, after which the uric acid concentration was determined by spectrophotometry.

#### 2.3.3. Amino Acid (AA) and Chitin Composition

To perform the AA determination, the feed and mealworm samples were prepared using a 22 h hydrolysis step in 6 N HCl at 112 °C under a nitrogen atmosphere. The AA in hydrolysate was determined by HPLC (Waters Alliance System with Waters 1525 Binary HPLC pump, Waters 2707 autosampler, and Waters 2475 multi λ fluorescence detector; Milford, MA, USA) after derivatization and according to the procedure described by Madrid et al. [18]. Tryptophan was not determined. 

For chitin estimation, glucosamine was determined following the method described for amino acids. Chitin estimation was performed according to the calculation proposed by Crespo et al. [19]. Briefly, it was considered that 215 g of glucosamine–HCl standard was equivalent to 179 g of glucosamine and that 221 g of chitin would yield 179 g of glucosamine due to deacetylation of the chitin molecule.

#### 2.3.4. Mineral Composition 

For the analysis of the mineral concentrations in the feeds, the ash samples were diluted in 0.6 N HNO_3_ solution and filtered. P was measured using the vanadate–molybdate method according to the official analytical method described in the Boletin Oficial del Estado [15], and the other minerals (Ca, Na, Mg, K, Fe, Mn, Cu, and Zn) were determined with an atomic absorption spectrophotometer (SOLAAR M series, Thermo Fisher Scientific, Waltham, MA, USA).

For mineral determination in the larvae, the samples were digested in a microwave digestion system (Milestone Ethos X Microwave, Sorisole, Italy) in the presence of HNO_3_. The minerals were determined by inductively coupled plasma–mass spectrometry (Agilent 7900 ICP–MS, Santa Clara, CA, USA) using the method of standard addition.

### 2.4. Gut Microbiota

#### 2.4.1. Gut Extraction

The insect guts (one insect per replicate) were extracted using the method of Wang and Zhang [11] with modifications. In brief, after sacrifice by freezing, the larvae were externally sterilized with 75% ethanol, being immersed for a total of 10 times. After that, they were immersed 10 times in sterilized water. A single insect was placed in a sterilized Petri dish, the anterior and posterior tips were removed, and the gut was pulled from the posterior end using sterilized forceps. The entire gut was deposited in an Eppendorf tube and frozen at −80 °C.

The samples were processed at the Genomics Platform of the Murcian Biosanitary Investigation Institute (Instituto Murciano de Investigación Biosanitaria (IMIB)) in the Health Sciences Campus of the University of Murcia (Murcia, Spain).

#### 2.4.2. DNA Extraction

DNA extraction was carried out using the Maxwell^®^ RSC PureFood GMO and Authentication Kit (Maxwell AS 3000, Promega, Madison, WI, USA). Then, the purity and concentration were determined by measuring the absorbance at 230, 260, and 280 nm (Infinite^®^200 PRO NanoQuant, Tecan, Mannedorf, Switzerland) and ensuring the optimum quality levels according to the absorbance ratios.

#### 2.4.3. Amplification, Library Preparation, and Sequencing

Bacterial identification was performed by sequencing the hypervariable regions of the 16S rRNA gene using two sets of primers that selectively amplify regions V2–4–8 and V3–6 and 7–9. 

The 16S rRNA gene was amplified using an Ion Torrent 16S Metagenomics kit (Thermo Fisher Scientific Inc., Warrington, UK). The PCR products were tested by 2% agarose gel electrophoresis, purified with AMPure^®^ XP Beads (Beckman Coulter Inc., Atlanta, GA, USA), and quantified using a Qubit^TM^ dsDNA HS Assay Kit (Invitrogen, Thermo Fisher Scientific Inc., Warrington, UK). 

Libraries were then prepared with an Ion Plus Fragment Library Kit (Ion Torrent, Thermo Fisher Scientific Inc., Warrington, UK), indexing each sample with Ion Xpress ™ Barcode Adapters (Ion Torrent, Thermo Fisher Scientific Inc., Warrington, UK) according to the manufacturer’s protocol. The library pool was then clonally amplified by emulsion PCR using ion sphere particles (ISPs). Template preparation was performed on the Ion OneTouch™ 2 (Ion Torrent, Thermo Fisher Scientific Inc., Warrington, UK). Finally, sequencing was performed using a 530 chip on an Ion S5™ (Ion Torrent, Thermo Fisher Scientific Inc., Warrington, UK).

#### 2.4.4. Bioinformatic Analysis

The data analysis stage was performed automatically using two software packages: Torrent Suite ™ v5.12.1 and Ion Reporter™ v5.16 with the 16S Metagenomics workflow module (Life Technologies, Grand Island, NY, USA). Clustering into operational taxonomic units (OTUs) and taxonomic assignment were performed based on the Basic Local Alignment Search Tool (BLAST) using two reference libraries, MicroSEQ^®^ 16S Reference Library v2013.1 and the Greengenes v13.5 database. Identification was accepted at the family, genus, and species level with sequence identities of >97%, >97%, and >99%, respectively, following the Clinical and Laboratory Standards Institute (CLSI) guidelines. Quantitative Insights Into Microbial Ecology (QIIME) software [20] was used to calculate alpha diversity.

For beta-diversity analysis, the vegan package in R was used, with analysis according to the Bray–Curtis distance matrix. The following tests were applied: analysis of similarity (ANOSIM) [21], multi-response permutation procedure (MRPP) [22], and non-parametric multivariate ANOVA (ADONIS) [23] with principal coordinate analysis (PCoA) performed using the Analyses of Phylogenetics and Evolution library. 

### 2.5. Performance and Digestibility Calculation

Feed intake and live body weight gain were used to calculate the feed conversion rate (FCR) and the efficiency of conversion of ingested food (ECI) [24] as follows:

$$\mathrm{FCR}=\frac{\mathrm{Feed}\;\mathrm{intake}\;\left(g\;\mathrm{DM}\right)}{\mathrm{Weight}\;\mathrm{gained}\;\left(g\;\mathrm{DM}\right)}$$

$$\mathrm{ECI}=\frac{\mathrm{Weight}\;\mathrm{gained}\left(g\;\mathrm{DM}\right)}{\mathrm{Feed}\;\mathrm{intake}\left(g\;\mathrm{DM}\right)}\times \;100$$

For the intake and digestibility determination, we calculated the total excreta of each replicate, assuming that uric acid excretion was constant for each diet [7]. Then, we calculated the total amount of refusal feed and excreta, using the amount of uric acid in the pure frass as a reference. Then, the DM digestibility coefficient (DMD) was determined using the following formula:

$$\mathrm{DMD}\%\;=\frac{\left(\mathrm{DM}\;\mathrm{ingested}-\mathrm{DM}\;\mathrm{excreted}\right)}{\mathrm{DM}\;\mathrm{ingested}}\times 100$$

### 2.6. Statistical Analysis

The data for the performance, digestibility, insect composition, and microbiota abundance were subjected to analysis of variance (ANOVA) with GLM procedures in IBM SPSS Statistics software (IBM Corporation, Armonk, NY, USA). Orthogonal polynomial contrasts were also used to determine the linear effect of the level of protein in the diet. Each plastic container was considered an experimental unit for the performance and digestibility data (*n* = 10 per treatment) and for the insect composition and microbiota abundance data, *n* = 5 per treatment. The Shapiro–Wilk test was used to establish the normality or non-normality of distribution. Tukey’s test was applied for post hoc comparison to evaluate the differences between means. The results were expressed as the least squares mean and standard error of the mean (SEM). Pearson’s correlation between the protein, starch, FND, and FAD diet content and DMD was also determined. The significance level was set at *p* ≤ 0.05.

## 3. Results

### 3.1. Performance and Digestibility 

The productive parameters and digestibility results are summarized in Table 3. The final individual weight and weight gain of the larvae were linearly higher for the HS-LP group compared to the other treatments (*p* < 0.001). The duration of the trial, evaluated in terms of first pupa emergence, was shorter for LS-HP and LS-HP compared to MS-MP (*p* < 0.001). In addition, the total intake increased linearly as the protein level in the diet increased (*p* < 0.001). Comparison of treatments showed that FCR was linearly the lowest and ECI linearly the highest in the HS-LP group (*p* = 0.001). A similar result was observed for DMD, being the highest for the HS-LP group (*p* < 0.001). In addition, DMD was positively correlated with the diet starch content (r = 0.748, *p* < 0.01) and negatively with the diet ADF content (r = –0.789, *p* < 0.01).

The amount of uric acid in the excreta linearly increased with the diet CP content (*p* < 0.001). Finally, the mortality rate was unaffected (*p* > 0.05) by the dietary treatment.

### 3.2. Chemical Composition of Insects

The dry matter, ash, and crude protein content of the larvae were influenced (*p* < 0.05) by the type of diet (Table 4). The dry matter and crude protein content were higher (*p* < 0.05) in mealworms reared on LS-HP and MS-MP, although the protein content of the MS-MP and HS-LP larvae did not differ. In addition, the ash content was significantly lower in mealworms reared on the LS-HP diet. The ether extract and chitin contents in the larvae were not influenced (*p* > 0.05) by the diet.

Table 5 lists the mineral composition of TM larvae depending on the rearing substrate. Except in the case of arsenic, which was higher (*p* < 0.05) in larvae reared with MS-MP and LS-HP diets based on agriculture by-products, there were no differences (*p* > 0.05) between mineral ratios.

Table 6 shows the amino acid composition of the TM larvae according to the rearing substrate. There were no statistically significant differences between the amino acid ratios regardless of the substrate.

### 3.3. Microbiota 

The number of sequences in the digestive tract samples of the TM larvae ranged from 161,719 to 59,607 in 15 samples, with a mean value of 110,863 sequences per sample. After OTU selection and accounting for chimeras, a total of 225 OTUs were obtained for the 15 samples. To calculate the richness and diversity indices, the sequences were normalized to 9412. The alpha diversity index results are shown in Table 7. The diet affected the Shannon and Simpson index of the TM larva gut microbiota (*p* = 0.05). The highest values were observed in the gut microbiota of larvae reared on LS-HP, which were not different from the gut microbiota of those reared on MS-MP.

Additionally, beta diversity was used to examine the dissimilarities in overall microbial community composition across the three diet types. Differences between bacterial communities were significant, as determined using ANOSIM (R = 0.415, *p* = 0.003). A PCoA plot based on the Bray–Curtis dissimilarity revealed that the HS-LP group was distinct from the LS-HP group (Figure S1). A significant difference was observed between the HS-LP and LS-HP groups (R = 0.796, *p* = 0.01), while no difference was observed between the HS-LP or LS-HP and MS-MP groups (R = 0.180, *p* = 0.092 and R = 0.240, *p* = 0.088, respectively). The differences between the diets as determined by ANOSIM were further supported by ADONIS (*p* = 0.024) and MRPP (*p* = 0.033).

The phyla of microbes identified in the digestive tracts of the TM larvae are shown in Table 8. A total of seven phyla were identified, among which 99.8% of the sequenced microbiota were included. The predominant phyla were Tenericutes, Firmicutes, and Proteobacteria, and all three were detected in the 15 analyzed digestive tract samples. In contrast, the minority phyla were Actinobacteria, Bacteroidetes, Fusobacteria, and Cyanobacteria. Proteobacteria and Firmicutes were the two in which the highest numbers of families and genera were detected. There were significant differences in the abundance of Tenericutes, Proteobacteria, and Cyanobacteria depending on the rearing substrate. The abundance of Tenericutes was higher (*p* < 0.01) in mealworms reared on the MS-MP and HS-LP diets. In contrast, the abundance of Proteobacteria and Cyanobacteria was markedly higher (*p* < 0.001 and 0.1, respectively) in insects reared on LS-HP.

Table 9 shows the most abundant families represented in the gut microbiota of TM larvae. The three most abundant families were Spiroplasmataceae, Moraxellaceae, and Lactobacillaceae. Spiroplasmataceae and Lactobacillaceae were detected in the 15 samples and Moraxellaceae in 13 samples. Members of the other identified families, Bacillaceae and Enterobacteriaceae, were found in 100% of the analyzed samples. Clostridiaceae and Bacteroidaceae, although present in low proportions (on average 0.6 and 0.3%, respectively), were detected in almost all of the analyzed samples (93%).

The abundance of members of Moraxellaceae, Nostocaceae, Leuconostocaceae, Staphylococcaceae, and Rhodobacteraceae families was higher (*p* < 0.05) in the mealworms reared on LS-HP. In contrast, Spiroplasmataceae was more abundant (*p* < 0.01) in the larvae of the HS-LP and MS-MP groups, and Lactobacillaceae in the larvae reared on HS-LP (*p* < 0.05).

In the TM microbiota, the three predominant genera were *Spiroplasma*, *Acinetobacter*, and *Bacillus* (Table S1). Both *Spiroplasma* and *Bacillus* were detected in 100% of the analyzed samples, while *Acinetobacter* was present in 12 samples. There were differences in abundance between *Spiroplasma*, *Lactobacillus*, *Staphylococcus,* and *Leuconostoc*. *Spiroplasma* levels were superior in mealworms reared on MS-MP, followed closely by the HS-LP group, in comparison with the LS-HP group (*p* < 0.05). *Lactobacillus* showed a higher abundance (*p* < 0.05) in the insects reared on the HS-LP diet. *Leuconostoc* and *Staphylococcus* showed a higher abundance (*p* < 0.05) in the mealworms reared on LS-HP.

## 4. Discussion 

The main objective of this study was to evaluate certain byproducts from the Mediterranean area as potential nutritional sources for rearing mealworms. Firstly, the accumulation of agricultural byproducts in this geographical area represents an environmental problem due to their polluting effect, as in the case of rice straw according to Viana et al. [25]. Secondly, most agricultural byproducts have a highly non-degradable fiber content, with a high content of lignocellulosic materials [26], which makes them poorly digestible by farm animals. The second objective was to evaluate the bioconversion role of TM, an insect species known for its versatile digestive system, capable even of degrading recalcitrant plastics [27], which makes them efficient at converting low-quality raw materials into biomass of high nutritional value [28,29,30]. In this way, a circular economy can be promoted in the context of new and emerging insect farming, as has already been highlighted [31]. However, this study is also justified by the need to determine the optimal balanced diets rather than simply using byproducts themselves, where the nutritional imbalances are considerable and will affect larval development. Due to this, the byproduct-based diets used had varying levels of protein, carbohydrates, fats, and fiber.

Regarding performance, we found that the higher-starch and lower-protein diet resulted in higher digestibility and better feed conversion efficiency but lower substrate intake and lower body weight gain. The high fiber content of the MS-MP and LS-HP diets led to lower digestibility. Consequently, in order to meet their energy requirements, mealworms ingested greater quantities of the substrate. Similarly, the level of dietary fiber could also have influenced the poor FCR of the high-fiber MS-MP and LS-HP diets in comparison to the HS-LP diet (1.67 and 1.58 versus 1.39 g/g, respectively). With diets based on byproducts (spent grains and beer yeast, bread and cookie remains, potato peelings, and maize distillers’ dried grains with solubles), Van Broekhoven et al. [7] reported higher FCR values and worse efficiency than in our study. They concluded that these diets may contain components that are difficult to digest or toxic to TM. Additionally, Oonincx and de Boer [32] use a cereal-based diet and obtained an FCR of 2.2, which is a poorer feed conversion performance compared to that in our study. It is noteworthy that the diets in our study did not comprise only starch and protein; consequently, other different compounds could have been responsible for the observed effects.

In the present study, the larvae reared on the LS-HP diet excreted more uric acid than those fed with the other diets. This could be related to an excess of protein in the diet, which is catabolized into uric acid—which we found in the excreta—which is similar to other animal species. Van Broekhoven et al. [7] reported the same observation, suggesting that mealworms can use this metabolic strategy to eliminate excess protein in their diet.

Regarding the dry matter digestibility coefficients, we did not find any scientific papers on diet digestibility in TM larvae. We found ADF content to be negatively correlated with digestibility, and a positive correlation between starch and digestibility. Despite this, the best-performing larvae in terms of FCR were those that had more fiber in their diet, which would show that, although these mealworms have a limited capacity to digest fiber, they have minimum requirements for the adequate functioning of their digestive system, as is the case in omnivorous monogastric animals. In agreement with our results, Li et al. [33] studied the fiber levels (0–20% crude fiber) in TM diets and concluded that a CF level between 5 and 10% is necessary for the larvae, as instars later reached optimal levels of growth, development, and respiration. Ruschioni et al. [30] studied the biological effects of TM diets based on olive byproducts, with the CF content ranging from 0.58 to 31.45%, and observed a negative effect when the CF content was greater than 16.77%. Morales-Ramos et al. [34], in a self-selection experiment with TM larvae using self-selected FND percentages ranging 22.52–34.94%, concluded that FND intake negatively impacted food assimilation, food conversion, and biomass gain. In contrast, Yang et al. [35] concluded that crop residues with a very high fiber content (such as rice straw, rice bran, or maize straw) support the vital activity of TM, which are effective at degrading lignin, hemicellulose, and cellulose.

The protein content of the insects obtained in this study was higher (45.96–52.46% DM) than that reported by Van Broekhoven et al. (45.1–48.6% DM) [7]. In addition, the diet composition was found to affect the insect composition, with more protein in the larvae fed the LS-HP diet that also had the highest CP content. This effect has also been noted by other authors, as reported below. Using byproduct-based diets, Ruschioni et al. [30] included different amounts of olive pomace in feeds for TM and found that as the dietary protein content increased, the protein content of the larvae increased. Alves et al. [36] included bocaiuva (*Acrocomia aculeata*) pulp flour in varying amounts in mealworm diets and observed that the larvae reared on the diet with the highest protein and lowest fat levels also had the highest protein and lowest fat content. Mancini et al. [29], using cereal byproduct-based diets, also observed that the protein content of the diet influenced the protein content of the TM and concluded that the TM body composition showed considerable plasticity in relation to diet.

On the other hand, Harsányi et al. [37] observed a low variation in the protein and fat content of the TM larvae despite the differences in dietary composition. Silva et al. [38] reported that the fat content of the diet did not strongly influence the fat content of the mealworms.

When the larvae are reared on a nutritionally balanced diet, the CP content of the diet should not affect the CP content in the larvae, and excess CP will be catabolized. Consequently, increased uric acid in the excreta will be observed [7]. Therefore, a higher crude protein content in the larvae could be due to a lower accumulation of fat promoted by diets poorer in energy sources and vice versa. This effect has also been observed in migratory locusts (*Locusta migratoria*) [39] and could be the cause of the variability reported by different authors. 

In contrast to our study, some authors have indicated that the amino acid content of TM was affected by diet [40]. This difference may be due to the different units used to express the amino acid content (per 100 g of DM). We expressed amino acid content per 100 g of protein and observed that the amino acid profile of TM did not depend on diet. 

The mineral composition was constant regardless of the diet. The levels of heavy metals (Cd, Pb, and As) in the TM larvae were found to be within the allowable ranges for feed according to EU legislation (EU Regulation 1275/2013) [41].

Several microbiota studies have been carried out on TM and products derived from them, focusing on the search for pathogenic microorganisms and aimed at providing the food safety necessary for their adequate consumption [42,43,44,45]. However, articles in which the gut microbiota are sequenced are scarcer and focus on the capacity to degrade certain kinds of plastic [9,27,46,47,48]. 

In our research, the most abundant and frequent phyla in TM gut microbiota were Tenericutes, Proteobacteria, and Firmicutes, similar to the reports of Jung et al. [49] on larvae reared in soil, of Garofalo et al. [43] on TM larvae purchased from a local market, and of Wang and Zhang [11] on TM larvae fed on wheat bran and various vegetables. Similar phyla have been observed in other insect species. Garofalo et al. [43] reported that in house cricket (*Acheta domesticus*), the most abundant phyla observed were Proteobacteria (42.6%), Firmicutes (34.0%), and Bacteroidetes (22.2%), and in *Locusta migratoria*, microbes were almost exclusively of the phylum Firmicutes (94.7%). In American cockroach (*Periplaneta americana*), an omnivorous cockroach species, the predominant phyla were Bacteroidetes, Firmicutes, and Proteobacteria [50]. On the other hand, in herbivorous, highly specialized species, such as domestic silkworm (*Bombyx mori*), the most abundant phyla were Cyanobacteria, Firmicutes, Proteobacteria, Bacteroidetes, and Actinobacteria [51]. In omnivorous insects, bacterial diversity in the gut is higher than in stenophagous (carnivorous and herbivorous) insects [52]; this shows that gut bacterial communities can be shaped by the host diet composition [11].

The most representative genus of Tenericutes is *Spiroplasma*, which is a commensal bacterium that establishes itself in the midgut of invertebrate hosts, in some cases showing pathogenic activity. Osimani et al. [44], studying yellow mealworms raised on sterilized wheat bran, observed more *Spiroplasma* in the hindgut and thus concluded that *Spiroplasma* may be heritable and co-evolve with *Tenebrio molitor*, or they may outcompete other bacterial species. 

Diet was shown to affect the gut microbiota profile of TM larvae. Thus, the phylum Tenericutes (which includes the Spiroplasmataceae family) was less dominant in the microbiota of larvae reared on the LS-HP diet, in which the phyla Proteobacteria (including the Moraxellaceae family) and Cyanobacteria (including the Nostocaceae family) were found to be dominant. At the genus level, *Spiroplasma* and *Lactobacillus* were significantly associated with a higher starch content, and the abundance of *Staphylococcus* and *Leuconostoc* was associated with a higher protein content in the diet. *Lactobacillus* is a sugar-fermenting bacterium in some Coleoptera insects [53]. Studying bran diets, Lou et al. [54] also observed increases in lactic acid bacteria (*Lactobacillus*), indicating that these bacteria could help maintain a stable gut environment, improve the distribution of gut microbes, and prevent the colonization of harmful bacteria.

The impact of diet on the gut microbiota is an accepted and widely studied phenomenon in higher species such as humans [55]. Although it is suggested that a diet based on insects has a major impact on their microbiota [56], there are few studies on the effect of the nutrient composition of this diet on TM gut microbiota. In *Hermetia illucens* larvae, Bruno et al. [57] observed an increase in the abundance of Proteobacteria in the gut when the CP content was increased by adding fish meal to the diet. 

## 5. Conclusions

In this study, dietary protein levels were found to have a great influence on TM development, but carbohydrates were also found to be important; therefore, special emphasis should be placed on the development of balanced diets. In conclusion, the larval growth, digestibility, composition, and gut microbiota of *Tenebrio molitor* depends on the diet they are administered, suggesting great potential for their rearing and production from byproducts, which is essential in determining the optimal conditions for possible industrial-scale rearing in the context of a circular economy.

## Figures and Tables

**Table 1:** Ingredients’ composition ^1^ of the byproducts used for diets for *Tenebrio molitor*.

	Courgette By-Product	Tigernut Pulp	Brewer’s Spent Grains	Brewer’s Yeast	Bread Remains	Rice Straw
Dry matter (DM %)	5.64	17.12	24.90	95.12	76.20	93.43
Composition (% DM)						
Ash	12.12	0.85	2.75	5.72	2.26	16.01
Crude Protein	23.01	5.60	27.26	48.48	13.40	2.85
Starch	0.0	19.01	2.64	0.0	78.75	0.0
Ether Extract	1.86	18.61	8.34	1.02	0.27	1.05
NDF ^2^	19.12	54.80	45.35	0.07	5.27	68.23
ADF ^3^	13.42	23.85	15.80	0.0	0.53	40.91
ADL ^4^	1.22	4.40	1.68	0.0	0.16	2.86

^1^ The analyzes of the chemical composition of ingredients were carried out according to the description indicated in Section 2.3. ^2^ NDF: neutral detergent fiber. ^3^ ADF: acid detergent fiber. ^4^ ADL: acid detergent lignin.

**Table 2:** Composition of the experimental diets for *Tenebrio molitor*.

	HS-LP ^1^	MS-MP	LS-HP
Ingredient composition (% as feed)			
Courgette by-product		30.00	25.00
Tiger nut pulp		5.00	5.00
Brewer’s grains		10.00	25.00
Bread remains	85.00	40.00	20.00
Brewer’s yeast	15.00	10.00	20.00
Rice straw		5.00	5.00
Total	100	100	100
Analyzed composition (% dry matter (DM)) ^2^			
Crude protein	18.51	21.03	26.34
Starch	61.10	29.81	20.01
Ether extract	2.80	5.85	7.12
Neutral detergent fiber	10.32	17.50	19.91
Acid detergent fiber	0.70	9.52	10.01
Acid detergent lignin	0.23	1.14	1.21
Macrominerals			
Ca	0.01	0.08	0.08
P	0.33	0.52	0.60
Na	0.67	0.29	0.20
Mg	0.04	0.14	0.15
K	0.50	1.91	1.75
Trace minerals (mg/100 g DM)			
Fe	2.92	4.84	4.07
Mn	0.27	1.28	1.79
Cu	0.35	0.42	1.08
Zn	3.67	5.29	6.89
Amino acids (% DM)			
Arginine	0.69	0.77	1.00
Histidine	0.40	0.37	0.47
Isoleucine	0.59	0.63	0.82
Leucine	0.98	1.05	1.39
Lysine	0.52	0.63	0.91
Methionine	0.61	0.65	0.78
Phenylalanine	0.81	0.70	0.91
Threonine	0.56	0.55	0.75
Valine	0.64	0.71	0.92
Alanine	0.54	0.88	1.16
Aspartic acid	0.74	1.17	1.60
Cysteine	1.13	1.13	1.33
Glycine	0.61	0.62	0.75
Glutamic acid	3.06	2.90	3.39
Proline	1.22	1.33	1.48
Serine	0.77	0.75	0.94
Tyrosine	0.43	0.42	0.59

^1^ HS-LP: diet containing a high level of starch and low level of protein; MS-MP: diet containing moderate starch and moderate crude protein amount; LS-HP: diet containing low starch and high crude protein amount. ^2^ The analyzes of the chemical composition of feeds were carried out according to the description indicated in Section 2.3.

**Table 3:** Productive parameters, digestibility, and fecal uric acid levels of *Tenebrio molitor* larvae reared on by-product-based diets.

	Diet ^1^			SEM ^2^	*p*-Value
	HS-LP	MS-MP	LS-HP		
Initial individual weight (mg)	0.60	0.65	0.69	0.079	0.898
Final individual weight (mg)	100.29 ^b^	112.99 ^b^	168.69 ^a^	2.483	<0.001 ***
Weight gain (mg)	99.69 ^b^	112.34 ^b^	168.00 ^a^	2.517	<0.001 ***
First pupae emergence (days)	88.90 ^b^	120.09 ^a^	91.00 ^b^	2.840	<0.001
Mortality (%)	15.90	13.63	14.54	1.775	0.871
Total intake (mg/larvae)	133.43 ^c^	207.43 ^b^	275.21 ^a^	5.746	<0.001 ***
Feed Conversion Ratio (g/g)	1.39 ^b^	1.67 ^a^	1.58 ^a^	0.026	0.001 **
Efficiency Conversion Ingested (%)	72.68 ^a^	60.37 ^b^	63.13 ^b^	1.208	0.001 **
Coefficient of DM ^3^ digestibility (%)	70.38 ^a^	49.63 ^c^	56.47 ^b^	1.012	<0.001 ***
Uric acid (mg/mg excreta)	0.01 ^c^	0.02 ^b^	0.03 ^a^	0.001	<0.001 ***

^1^ HS-LP: diet containing a high level of starch and low level of protein; MS-MP: diet containing moderate starch and moderate crude protein amount; LS-HP: diet containing low starch and high crude protein amount. ^2^ SEM: Standard error of the mean (*n* = 10). ^abc^ Means values followed by different letters in the same row are different (*p* < 0.05); **, linear effect (*p* < 0.01); ***, linear effect (*p* < 0.001). ^3^ DM: dry matter.

**Table 4:** Effects of the diets on the chemical composition of *Tenebrio molitor* larvae (*n* = 5).

	Diet ^1^			SEM ^2^	*p*-Value
	HS-LP	MS-MP	LS-HP		
Dry matter (DM, %)	34.32 ^b^	41.78 ^a^	44.21 ^a^	1.219	0.007
Composition (% DM)					
Ash	3.83 ^a^	3.72 ^a^	3.48 ^b^	0.014	0.004
Crude protein	45.96 ^b^	49.07 ^ab^	52.46 ^a^	0.551	0.039
Ether extract	33.92	28.93	27.30	1.130	0.186
Chitin	3.06	3.78	4.82	0.583	0.540

^1^ HS-LP: diet containing a high level of starch and low level of protein; MS-MP: diet containing moderate starch and moderate crude protein amount; LS-HP: diet containing low starch and high crude protein amount. ^2^ SEM: Standard error of the mean (*n* = 5). ^ab^ Means values followed by different letters in the same row are different (*p* < 0.05).

**Table 5:** Effects of the diets on the mineral composition of *Tenebrio molitor* larvae.

	Diet ^1^			SEM ^2^	*p*-Value
	HS-LP	MS-MP	LS-HP		
Macrominerals (% DM)					
Ca	0.06	0.09	0.11	0.008	0.167
P	0.72	0.83	0.86	0.043	0.458
Na	0.29	0.22	0.19	0.018	0.236
Mg	0.16	0.21	0.24	0.013	0.183
K	1.00	1.22	1.12	0.052	0.375
Trace elements (mg/100 g DM)					
Fe	3.65	4.15	3.95	0.239	0.724
Cu	0.86	1.37	1.35	0.057	0.059
Zn	9.44	13.10	17.00	0.811	0.071
Al	0.11	0.08	0.06	0.029	0.763
Cr	0.01	0.01	0.01	0.001	0.402
Mn	0.49	0.84	1.06	0.055	0.056
Co	0.01	0.01	0.01	0.001	0.140
Ni	0.03	0.03	0.04	0.003	0.901
Ba	0.05	0.25	0.05	0.070	0.476
As	0.01 ^b^	0.06 ^a^	0.06 ^a^	0.001	0.017
Cd	0.04	0.04	0.04	0.001	0.589
Pb	0.40	0. 05	0. 11	0.007	0.209

^1^ HS-LP: diet containing a high level of starch and low level of protein; MS-MP: diet containing moderate starch and moderate crude protein amount; LS-HP: diet containing low starch and high crude protein amount. ^2^ SEM, Standard error of the mean (*n* = 5); ^ab^, means values followed by different letters in the same row are different (*p* < 0.05).

**Table 6:** Effects of the diets on the amino acid composition of *Tenebrio molitor* larvae (expressed as crude protein percentage).

	Diet ^1^			SEM ^2^	*p*-Value
	HS-LP	MS-MP	LS-HP		
Essential amino acids					
Arginine	5.22	5.96	5.12	0.092	0.061
Histidine	3.11	3.41	2.90	0.149	0.473
Isoleucine	4.80	4.89	4.48	0.050	0.085
Leucine	7.03	7.25	6.96	0.060	0.273
Lysine	4.43	4.34	5.67	0.318	0.305
Methionine	2.97	2.26	2.94	0.245	0.494
Phenylalanine	4.14	4.27	3.76	0.292	0.780
Threonine	4.53	4.57	4.04	0.183	0.514
Valine	6.36	6.66	6.20	0.088	0.236
No essential amino acids					
Alanine	7.97	7.94	7.58	0.099	0.333
Aspartic acid	5.83	6.01	7.94	0.211	0.054
Cysteine	8.08	6.80	8.24	0.662	0.664
Glycine	5.69	5.88	5.17	0.116	0.176
Glutamic acid	10.06	10.20	11.59	0.241	0.137
Proline	8.45	7.14	5.79	0.427	0.178
Serine	5.00	5.22	4.85	0.104	0.437
Tyrosine	6.36	7.21	6.78	0.327	0.619

^1^ HS-LP: diet containing a high level of starch and low level of protein; MS-MP: diet containing moderate starch and moderate crude protein amount; LS-HP: diet containing low starch and high crude protein amount. ^2^ SEM, Standard error of the mean (*n* = 5).

**Table 7:** Effects of the diets on *Alpha diversity* microbiota of *Tenebrio molitor* larvae.

	Diet ^1^			SEM ^2^	*p*-Value
	HS-LP	MS-MP	LS-HP		
Chao1 index	18.40	22.24	28.58	2.001	0.154
Shannon index	1.34 ^b^	1.78 ^ab^	2.71 ^a^	0.204	0.050
Simpson index	0.40 ^b^	0.51 ^ab^	0.77 ^a^	0.056	0.050

^1^ HS-LP: diet containing a high level of starch and low level of protein; MS-MP: diet containing moderate starch and moderate crude protein amount; LS-HP: diet containing low starch and high crude protein amount. ^2^ SEM: Standard error of the mean (*n* = 5). ^ab^ Means values followed by different letters in the same row are different (*p* < 0.05).

**Table 8:** Effect of diets on the abundance (%) of the most frequent phyla in the microbiota of *Tenebrio molitor* larvae.

	Diet ^1^			SEM ^2^	*p*-Value
	HS-LP	MS-MP	LS-HP		
Tenericutes	67.88 ^a^	70.08 ^a^	13.41 ^b^	6.645	0.007
Proteobacteria	6.04 ^b^	17.99 ^b^	42.84 ^a^	2.569	0.000
Firmicutes	23.62	7.89	28.71	4.731	0.222
Cyanobacteria	1.86 ^b^	1.23 ^b^	8.46 ^a^	0.754	0.004
Fusobacteria	0.04	2.09	5.23	2.051	0.582
Bacteroidetes	0.33	0.51	0.71	0.159	0.614
Actinobacteria	0.23	0.01	0.30	0.085	0.357

^1^ HS-LP: diet containing a high level of starch and low level of protein; MS-MP: diet containing moderate starch and moderate crude protein amount; LS-HP: diet containing low starch and high crude protein amount. ^2^ SEM, Standard error of the mean (*n* = 5). ^ab^ Means values followed by different letters in the same row are different (*p* < 0.05).

**Table 9:** Effect of diets on the abundance (%) of the 15 most frequent families in the microbiota of *Tenebrio molitor* larvae.

	Diet ^1^			SEM ^2^	*p*-Value
	HS-LP	MS-MP	LS-HP		
Spiroplasmataceae	67.84 ^a^	69.83 ^a^	13.41 ^b^	6.620	0.007
Moraxellaceae	1.33 ^b^	8.74 ^b^	30.75 ^a^	3.339	0.010
Lactobacillaceae	14.87 ^a^	0.11 ^b^	0.16 ^b^	2.317	0.044
Bacillaceae	5.95	4.75	11.03	1.742	0.313
Enterobacteriaceae	4.21	6.25	8.84	1.234	0.332
Nostocaceae	1.79 ^b^	1.22 ^b^	7.70 ^a^	0.722	0.006
Streptococcaceae	0.27	0.15	11.90	2.888	0.191
Fusobacteriaceae	0.04	2.09	5.23	2.051	0.582
Paenibacillaceae	0.32	1.23	2.11	0.407	0.235
Leuconostocaceae	0.50 ^b^	0.14 ^b^	1.88 ^a^	0.205	0.012
Staphylococcaceae	0.16 ^b^	0.20 ^b^	0.90 ^a^	0.083	0.006
Rhodobacteraceae	0.06 ^b^	0.21 ^b^	1.19 ^a^	0.160	0.028
Clostridiaceae	0.65	0.90	0.34	0.208	0.548
Bacteroidaceae	0.30	0.32	0.38	0.063	0.878
Xanthomonadaceae	0.00	1.08	0.04	0.330	0.367

^1^ HS-LP: diet containing a high level of starch and low level of protein; MS-MP: diet containing moderate starch and moderate crude protein amount; LS-HP: diet containing low starch and high crude protein amount. ^2^ SEM, Standard error of the mean (*n* = 5). ^ab^ Means values followed by different letters in the same row are different (*p* < 0.05).

## Data Availability

Not applicable.

## Supplementary Materials

The following supporting information can be downloaded at: https://www.mdpi.com/article/10.3390/insects13040323/s1, Figure S1: Principal coordinate analysis (PCoA) of the bacterial community structures of the *Tenebrio molitor* gut microbiota of the three diets. The PCoA plots were constructed according to Bray–Curtis dissimilarity (*n* = 5). The percentage of variation explained by the PCoA1 and PCoA2 plots are 36.7% and 18.5%, respectively. HS-LP: diet containing a high level of starch and a low level of protein; MS-MP: diet containing a moderate amount of starch and moderate crude protein; LS-HP: diet containing a low amount of starch and a high amount of crude protein; Table S1: Effect of diets on the abundance (%) of the 15 most represented genera in the microbiota of *Tenebrio molitor* larvae.
