# Supplementary material for: Effects of Agro-Industrial Byproduct-Based Diets on the Growth Performance, Digestibility, Nutritional and Microbiota Composition of Mealworm (Tenebrio molitor L.)
Source: Insects. 2022 Mar 25;13(4):323. doi: 10.3390/insects13040323 (PMC9027437; doi:10.3390/insects13040323)
Supplement: Supplementary file 1 [file insects-13-00323-s001.zip › Supplementary materials.pdf]

insects

Supplementary Materials

Effects of agro-industrial byproduct-based diets on the growth performance, digestibility, and nutritional and microbiota composition of mealworm (*Tenebrio molitor* L.)

Ana Montalbán, Cristian Jesús Sánchez, Fuensanta Hernández, Achille Schiavone, Josefa Madrid, Silvia Martínez-Miró

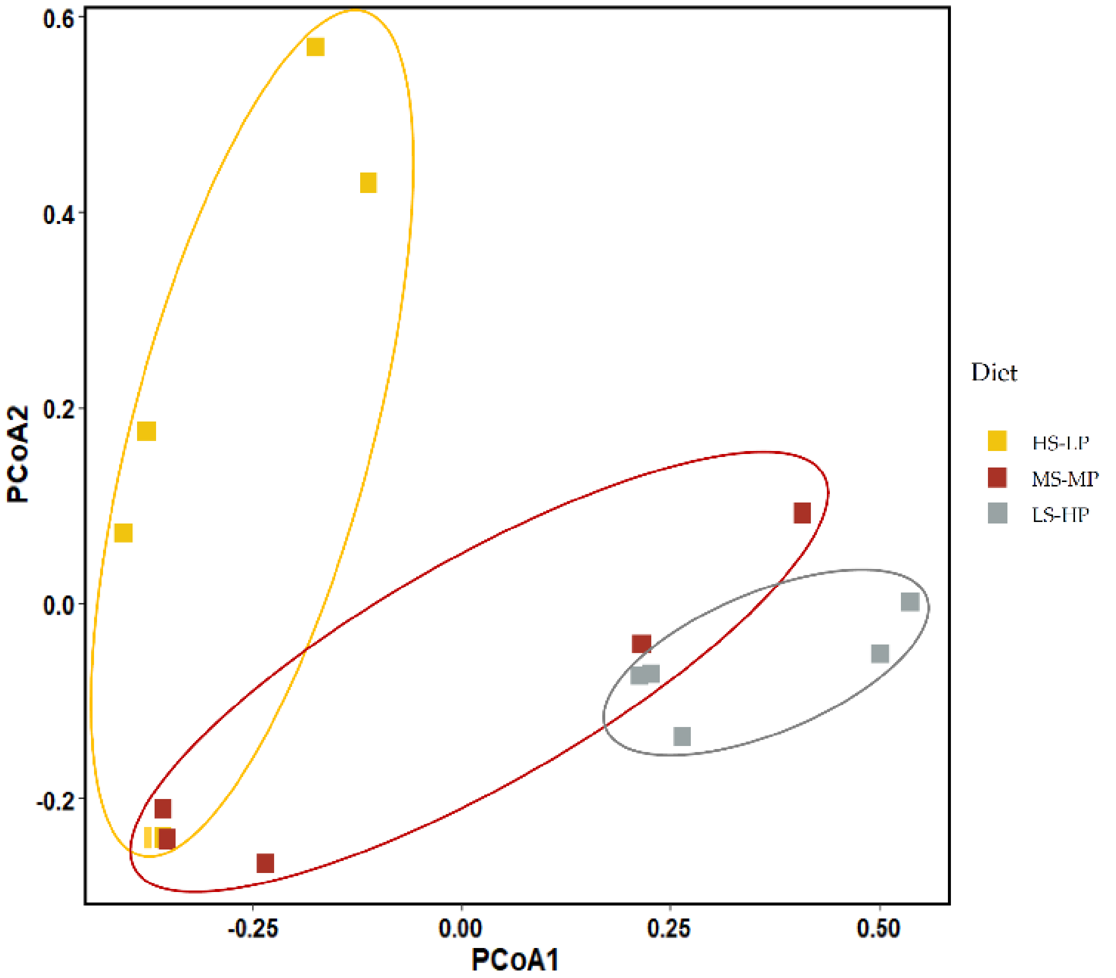

Figure S1. Principal coordinate analysis (PCoA) of the bacterial community structures of the *Tenebrio molitor* gut microbiota of the three diets. The PCoA plots were constructed according to Bray–Curtis dissimilarity (n = 5). The percentage of variation explained by the PCoA1 and PCoA2 plots are 36.7% and 18.5%, respectively. HS-LP: diet containing a high level of starch and a low level of protein; MS-MP: diet containing a moderate amount of starch and moderate crude protein; LS-HP: diet containing a low amount of starch and a high amount of crude protein.

insects

MDPI

Table S1. Effect of diets on the abundance (%) of the 15 most represented genera in the microbiota of *Tenebrio molitor* larvae.

	Diet [1]				
	HS-LP	MS-MP	LS-HP	SEM [2]	P-value
Spiroplasma	60.97 [a]	64.51 [a]	9.33 [b]	6.986	0.012
Acinetobacter	2.50	12.27	34.29	5.719	0.103
Bacillus	12.64	6.14	18.26	3.802	0.445
Cetobacterium	0.09	4.00	7.46	2.998	0.608
Plesiomonas	0.31	4.63	5.79	1.802	0.455
Pediococcus	10.34	0.02	0.03	3.158	0.356
Lactococcus	0.28	0.00	9.33	2.131	0.158
Brevibacillus	0.68	1.71	4.06	0.816	0.249
Lactobacillus	5.43 [a]	0.06 [b]	0.28 [b]	0.753	0.028
Clostridium	1.39	1.70	0.15	0.405	0.276
Weissella	1.05	0.14	1.98	0.397	0.205
Staphylococcus	0.35 [b]	0.28 [b]	1.67 [a]	0.231	0.049
Bacteroides	0.64	0.61	0.68	0.133	0.974
Leuconostoc	0.03 [b]	0.07 [b]	1.63 [a]	0.243	0.031
Aeromonas	0.04	0.89	0.32	0.204	0.286

[1] HS-LP: diet containing a high level of starch and low level of protein; MS-MP: diet containing moderate starch and moderate crude protein amount; LS-HP: diet containing low starch and high crude protein amount. [2] SEM, Standard error of the mean ($n = 5$). [ab] Means values followed by different letters in the same row are different ($p < 0.05$).
